# Sensory guided selection criteria for breeding consumer-preferred sweetpotatoes in Uganda

**DOI:** 10.1016/j.foodqual.2022.104628

**Published:** 2022-10

**Authors:** Mariam Nakitto, Suzanne D. Johanningsmeier, Mukani Moyo, Christophe Bugaud, Henriette de Kock, Layal Dahdouh, Nelly Forestier-Chiron, Julien Ricci, Elizabeth Khakasa, Reuben T. Ssali, Christian Mestres, Tawanda Muzhingi

**Affiliations:** a International Potato Center (CIP-SSA), Plot 47 Ntinda II Road, PO Box 22247, Kampala, Uganda; b Department of Consumer and Food Sciences, University of Pretoria, Private bag X20, Hatfield, Pretoria, South Africa; c United States Department of Agriculture, Agricultural Research Service, Southeast Area, Food Science and Market Quality & Handling Research Unit, Raleigh, NC 27695, USA; d International Potato Center (CIP-SSA Regional Office), PO Box 25171, Nairobi, Kenya; e CIRAD, UMR QUALISUD, 73 avenue J.F. Breton, F-34398 Montpellier Cedex 5, France; f QualiSud, Univ Montpellier, CIRAD, Montpellier SupAgro, Univ Avigon, Univ La Réunion, Montpellier, France; g National Agriculture Research Laboratories, PO Box 7065, Kawanda – Senge Road, Kampala, Uganda

**Keywords:** Descriptive sensory analysis, Sweetpotato lexicon, Sweetpotato breeding, *Ipomoea batatas*, Penalty analysis, Sweet potato

## Abstract

Prioritizing sensory attributes and consumer evaluation early in breeding trials to screen for end-user preferred traits could improve adoption rates of released genotypes. In this study, a lexicon and protocol for descriptive sensory analysis (DSA) was established for sweetpotato and used to validate an instrumental texture method for which critical values for consumer preference were set. The study comprised several phases: lexicon development during a 4-day workshop; 3-day intensive panel training; follow-up virtual training, evaluation of 12 advanced genotypes and 101 additional samples from two trials in 2021 by DSA and instrumental texture analysis using TPA double compression; and DSA, instrumental texture analysis and consumer acceptability tests on 7 genotypes in on-farm trials. The established sweetpotato lexicon comprising 27 sensory attributes enabled characterization and differentiation of genotypes by sensory profiles. Significant correlation was found between sensory firmness by hand and mouth with TPA peak positive force (r = 0.695 and r = 0.648, respectively) and positive area (r = 0.748, r = 0.715, respectively). D20, NAROSPOT 1, NASPOT 8, and Umbrella were the most liked genotypes in on-farm trials (overall liking = 7). An average peak positive force of 3700 gf was proposed as a minimum texture value for screening sweetpotato genotypes, since it corresponded with at least 46 % of consumers perceiving sweetpotatoes as just-about-right in firmness and a minimum overall liking of 6 on average. Combining DSA with instrumental texture analysis facilitates efficient screening of genotypes in sweetpotato breeding programs.

## 1 Introduction

Roots, tubers, and bananas are the main crops for nutrition and food security in many low-income African countries, including Uganda. Cassava was affected by cassava mosaic disease in the 1990s, while bananas succumbed to banana wilt disease at the beginning of the 21st century (Kagezi et al., 2006), which led to the emergence of sweetpotato (*Ipomea batatas* L.) as an important food security crop for sustaining the population against imminent hunger. Breeding programs have enhanced the crop by developing varieties with superior agronomic traits and resistance to several environmental stress factors, pests, diseases (Mwanga et al., 2011, Mwanga et al., 2016), and optimized nutritional composition (Gurmu et al., 2017, Low et al., 2017). Specifically, several orange-fleshed sweetpotato varieties that are high in carotenoids have been released (Ssemakula, et al., 2014). Some varieties are being biofortified with minerals such as zinc and iron. Unfortunately, the full benefit of these efforts is often challenged by poor adoption among the population due to low consumer acceptance.

Sensory characteristics of food are critical to consumer food choice and acceptability (Maina, 2018). Consumers of boiled and steamed sweetpotato in Uganda have expressed a preference for varieties that are sweet, dry, and mealy but not fibrous (Mwanga et al., 2021). Regardless, the process of breeding sweetpotato proceeds with minimal consideration for sensory attributes until shortly before variety release when hedonic evaluation is conducted (Ssemakula et al., 2014). Making sensory attributes among the main criteria for selection earlier on in the process could support the breeding of sweetpotatoes with consumer-preferred traits. To achieve this, steps should be taken to holistically understand the sensory characteristics of sweetpotato using descriptive sensory analysis, and then identify the genetic factors that influence them. Descriptive sensory analysis gives detailed and reliable information about the qualitative attributes of a product (Joanna et al., 2019) thus providing a basis for understanding acceptability. There are currently no standard protocols in place to guide descriptive sensory analysis for sweetpotato breeding in Uganda.

There are several critical aspects to conducting descriptive sensory analysis correctly. Usually, descriptive sensory analysis starts with a group of candidate panelists being recruited and screened for sensory acuity and availability (de Kock and Magano, 2020, ISO, 2005). Afterwards, the selected panel generates a lexicon for the products to be evaluated. They are then trained on using the lexicon to ensure reliable results. Besides the analytical ability of the panelists, the environment in which samples are evaluated is an important aspect of sensory analysis. The recommendation for classic descriptive sensory analysis is using sensory booths in a controlled laboratory environment (ISO, 2005). However, such facilities are not always available, expensive to construct, and therefore not an option in case of economic limitations. Furthermore, the recent COVID-19 pandemic restrictions complicated panelists’ access to laboratories requiring innovative applications. While the alternative of home use tests has been explored for consumer sensory analysis of sweetpotato (Moyo et al., 2021), home use test protocols for descriptive sensory analysis was lacking.

Breeding programs are tasked with screening hundreds of genotypes per season. While use of sensory panels and consumer acceptability tests are the most accurate measures for human sensory perception and liking (Meilgaard et al., 2007), time and other resources remain a challenge. High throughput instrumental methods which can be used to assess a large number of samples in a short time could facilitate the screening process if these measurements are correlated with human assessment of sensory attributes that are linked with end-user preferences. Texture (specifically, the perceived firmness) is a key sensory attribute that influences consumer acceptance of steamed or boiled sweetpotato (Mwanga et al., 2021). Earlier studies showed a significant relationship between instrumental and sensory panel assessment of steamed sweetpotato (Truong et al., 1997). More recently, an instrumental method for evaluating firmness of boiled sweetpotato was applied to determine texture differences among genetic variants (Banda et al., 2021, Banda et al., 2021). However, the methods used in the latter study were not specifically related to consumer preferences for texture.

The objectives of this study were 1) to develop a lexicon and protocol for evaluation of sweetpotato by a trained descriptive sensory analysis panel and 2) to validate a high throughput instrumental texture method to establish critical values related to consumer preference for application in sensory-guided sweetpotato breeding programs.

## 2 Material and methods

Ethical approval for the involvement of human subjects in this study was granted by Makerere University School of Social Sciences Research Ethics Committee (MAKSSREC 12.19.364), Centre de Coopération Internationale en Recherche Agronomique pour le Développement (French Agricultural Research Centre for International Development), CIRAD, Ethics committee, and retrospectively by the Faculty of Natural and Agricultural Sciences, University of Pretoria (NAS 236/2021).

### 2.1 Sweetpotato samples

Sweetpotato roots of various genotypes were obtained from the ongoing International Potato Center (CIP) breeding trials in various locations in Uganda, while some were obtained from farmers in the same areas. Roots sourced from the open market usually have a high variation within them and these were avoided. Many genotypes were used for lexicon development and sensory panel training in phases 1–3 (Table S.1 in Supplementary Material) while roots of 12 diverse sweetpotato genotypes of Development and Delivery of Biofortified Crops at Scale (DDBIO) multi-location advanced field trial planted in 2020 under a collaboration between the National Agricultural Research Organization (NARO) and CIP were used for the sensory and instrumental assessments in phase 4. Five of these 12 advanced trial genotypes were assessed by untrained consumers for acceptability as part of a pilot study to develop correlations between consumer liking, descriptive sensory analysis and instrumental texture. The subset of samples was selected based on the number of roots available and variation in flesh colors. Moderately sized sweetpotato roots according to the size distribution of the harvest with no visible damage were used in all cases (Porras et al., 2014).

In phase 5, sweetpotato roots were obtained from DDBIO multi-location advanced field trial planted in 2021 in five locations. The trial comprised 15 genotypes including 10 test clones (1.44, D11, D15, D20, D26, NKB3, NKB105, S36, S47 and S97) and 5 checks (Ejumula, New Kawogo, NASPOT 8, NASPOT 10O, NASPOT 11). Test clones are genotypes being studied for selection and potential release as new varieties while checks are released or local varieties of known agronomic performance. This multi-location trial included clones of the 12 genotypes harvested in 2020 under the same trial. In total, 61 unique samples were studied. Another set of sweetpotato roots representing 40 clones, 7 of which were among the raw material for making a new generation of genetic improvements referred to as parents (Beauregard, CEMSA 74–228, Ejumula, NAROSPOT 1, NASPOT 8, Silk Omuyaka and Tanzania), were obtained from Mwanga Diversity Panel (MDP) population planted in 2021. Descriptive sensory profiles by the trained panel and instrumental texture measures collected from these materials were used to establish relationships between sensory texture and instrumental texture parameters.

Consumer sensory analysis was conducted with 106 consumers with 7 genotypes planted as part of the on-farm trials in phase 6. The genotypes included two checks, NASPOT 8 and NAROSPOT 1; three test clones, D20, NKB3, NKB105; and two local clones, Muwulu Aduduma and Umbrella. On-farm trials are part of the final steps in a breeding cycle and were designed as participatory plant breeding where farmers are involved in breeding selections.

### 2.2 The trained sensory panel

Twenty-one individuals (11 men and 10 women; researchers and technicians) working at the National Agricultural Research Organization (NARO) in Uganda that had completed 50 h of prior sensory training with other roots, tubers and banana crops were recruited by the Food Biosciences and Agribusiness Program for the sensory panel in 2019. Participants were informed about the objectives and provided consent during the first training session. Candidates participated in phase 1, a first workshop of lexicon development (August 2019), with 10 continuing with the training that followed in phase 2 (October 2019). Nine panelists attended phase 3, a virtual training and analyzed sweetpotato samples in the office setting (July 2020).

#### 2.2.1 Phase 1 and 2: Lexicon development and initial panel training sessions

A draft lexicon was developed with 21 participants during a 4-day training workshop. The panel tasted 7 genotypes of contrasting sensory characteristics (Table S.1 in Supplementary Materials). A follow up session, facilitated by the sensory research leader, was held to discuss descriptive terms and to reach consensus among participants (Swegarden et al., 2019).

Once the list of descriptive terms was drafted, reference products were identified and presented to the panel. This step helped to reduce the number of attributes in the draft lexicon and to create descriptions for the attributes. Definitions for selected attributes were developed with reference to literature (Meilgaard et al., 2007, ISO, 2008) and modified as needed. Since the panelists came from various professional backgrounds, an easy-to-understand simplified version of each definition was created. Intensity scales anchored with verbal expressions were selected for each attribute and a workflow to guide sensory assessment was developed.

The lexicon was further refined and used to train 10 panelists during a 3-day workshop in phase 2. Here, participants evaluated 8 sweetpotato genotypes (Table S.1 in Supplementary Materials) and results were used to monitor panel performance and facilitate further training. Follow-up discussions at this stage facilitated by the panel leader led to finalizing the lexicon.

During the workshops, sensory analysis was conducted in a sensory laboratory with individual evaluation booths separate from the preparation area. Panelists evaluated samples and recorded their scores on a paper ballot. They were also provided with a document summarizing the workflow, attributes, definitions, and scales for reference. Drinking water and slices of fresh cucumber were provided as palate cleansers for use before and between samples.

#### 2.2.2 Phase 3: Virtual panel training during COVID 19 pandemic and sample evaluations in office settings

The panel received additional training evaluating various sweetpotato genotypes for 6 days in February 2020. Shortly thereafter, work with the sensory panel was challenged by the COVID 19 pandemic. Under these circumstances, home and office settings were identified as alternative sites for conducting the sensory tests.

The panel was divided into two groups and participated in one 3-hour virtual training session held via Microsoft Teams. Members who would be evaluating samples from home attended a special training session on how to prepare samples. Data were collected virtually using Compusense Cloud software (Academic Consortium, Compusense Cloud, Compusense Inc., Guelph, ON, Canada).

Four members of the panel were trained to prepare and evaluate the samples at their individual homes using cookware available in their kitchens. Raw sweetpotato roots were delivered to the panelists’ homes in labelled paper bags. However, the samples prepared at home were variably cooked (often undercooked), perhaps because they did not have the steaming pots which the research team uses in the laboratory. As a result, there were many cases where samples could not be evaluated. Therefore, data from this group were excluded.

#### 2.2.3 Phases 4, 5 and 6: Exploring and developing relationships between sensory firmness and instrumental texture parameters, and sensory and instrumental texture analysis of on-farm trials

In April and May 2021, 13 participants attended a 7-day training workshop where they were trained on the terms in the lexicon and how to conduct sensory evaluation of steamed sweetpotato. Among them, 12 trained panelists participated in descriptive sensory profiling of 12 advanced genotypes from DDBIO advanced field trial planted in 2020. From October to early November 2021, they participated in sensory profiling of 40 genotypes from the MDP trial population, and then from late November to December, all 13 panelists evaluated 61 genotypes from the DDBIO multi-location advanced field trial planted in 2021. In February 2022, a 4-day training, attended by trained panelists and 3 new participants, was held and 12 panelists evaluated genotypes used in on-farm trials.

#### 2.2.4 Preparation and presentation of samples for descriptive sensory analysis by the trained panel

Sample preparation evolved throughout the panel training phases. The roots were prepared in a central kitchen. During lexicon development (phase 1), sweetpotatoes were prepared following the commonly used local method where roots are peeled, wrapped in banana leaves, and steamed for 1 h in a saucepan as detailed in Mwanga et al. (2021) over gas. Several roots of the same genotype constituting a sample were wrapped separately and assigned a unique colored identification string. Despite the advantage of cultural compatibility, it was difficult to control variation in the cooked texture of the roots resulting from differences in their shape and size. As a result, the cooking method was revised for the next phases of training and the advanced trial assessment.

From phase 2 onwards, sweetpotato roots were prepared by cutting out a 7 cm portion (Figure S.1 in Supplementary Materials) weighing 160 – 240 g before peeling. The number of 7 cm portions prepared was equal to the number of respondents. The portions were peeled and placed upright to steam in single tiered steaming pots (Korkmaz Perla Cous Cous Cookware Set A152, Korkmaz Dis Ticaret Ltd, Turkey) with 2000 ml of water in the bottom pan for 1 h. The steamer layer was lined with a banana leaf on which the portions were placed, covered with another banana leaf and then the lid.

At service, the ends of the cooked 7 cm portions were cut off before wrapping each piece in aluminum foil. Wrapped samples were labelled with randomly assigned 3-digit codes and presented monadically to panelists. Samples were cooked in the same order as they were served at 20 – 30 min intervals. Once ready, a sample was served as soon as possible such that panelists tasted the samples at a temperature ranging from 50 to 60 °C. From phase 2 onwards, panelists evaluated samples in several sessions which consisted of three or four samples per session. Selected genotypes were served in duplicate across each sample tasting phase (Table S.1 in Supplementary Materials).

### 2.3 Instrumental texture analysis of sweetpotato

During preliminary experiments, various sample preparation methods, probes and program settings were compared to identify a method which produced the most repeatable and discriminative results (Nakitto et al. 2021). Following the selected method, 3 representative roots were selected from each genotype for instrumental texture analysis. Three pieces of 3x3x2.5 cm were cut from each root and placed on the steamer layer in a steaming pot (see 2.2.3) matted by a layer of banana leaf. The pieces were steamed for 35 min (from when the pot was placed on the gas fire). Afterwards, the pieces were carefully trimmed on either end to remove a slippery layer resulting from amylose leaching leaving a 3x3x2 cm piece. The texture of each piece at room temperature (20 – 25 °C) was analysed using a TA-XT texture analyzer (Stable Macro Systems, Godalming, UK) with 10 kg load cell, following a texture profile analysis (TPA) procedure adapted from Truong et al. (1997) and Banda et al. (2021a). In our method, a 60 mm diameter compression plate probe moving at a speed of 100 mm/min compressed the sample (2 cm vertical height) for 5 s (8.35 mm distance), resting for 5 s in between compressions (see Figure S.2 in Supplementary Materials). Truong had previously used the same crosshead speed but a higher (75 %) compression. In preliminary experiments, a lower compression of 25 % was found most suitable for the wide range of sweetpotato texture in the Ugandan breeding program.

The parameters that were recorded from the resulting curves were the peak positive forces and the positive areas of the two curves (Figure S.2 in Supplementary Materials). Based on experiments conducted when establishing the method, these parameters were reliable and discriminative (Nakitto et al. 2021). During these experiments it was also observed that there was a high variation in other instrumental texture parameters such as negative area. Regarding secondary parameters, adhesiveness was not calculated since there was a high variation in readings of negative area. Cohesiveness was calculated as the ratio of area under the second curve to area under the first curve, and gumminess was calculated as the product of peak force in the first compression and cohesiveness.

#### 2.3.1 Evaluation of dry matter of raw sweetpotato samples

To evaluate the dry matter of sweetpotato genotypes, 2 g of thinly sliced raw sweetpotato roots from each genotype were accurately weighed into a moisture dish in triplicate and the dry matter was evaluated following the method described by Adesokan, Alamu and Maziya Dixon (2020).

### 2.4 Consumer evaluation of sweetpotato

#### 2.4.1 Consumer respondents and questionnaire

During phase 4, 23 consumers of sweetpotato were invited to evaluate five (NASPOT 8, NASPOT 10O, NASPOT 11, NKB3 and S47) of the 12 genotypes from the DDBIO advanced field trial (Table S.1 in Supplementary Materials) planted in 2020 as part of a pilot study to design a consumer sensory analysis study. The subset of samples was selected based on the number of damage free roots available from the harvest and variation in flesh colors. The participants were recruited by the local leaders and included adult men and women consumers of sweetpotato. The number of participants were limited to the number of roots available for evaluation and number that the interviewers could manage given the curfew limitation of the COVID period.

Trained interviewers obtained consent from the participants in English or a local language where necessary. The interviewers then administered a questionnaire and entered responses via Compusense software. All samples were prepared as described in 2.2.4 and presented monadically to respondents who evaluated each sample once. All personal information that was collected was discretely stored by the research team. The full length of the questionnaire is presented as Appendix S.1 in Supplementary Materials.

Most of the respondents were men (56.5 %) (Table S.2 in Supplementary Materials) and working in the informal sector (43.5 %). The age range of the participants was 19 to 48 years, with an average of 31 years. Most of the respondents ate sweetpotato for lunch (78.3 %) several times a week (52.2 %).

Following this pilot study, the questionnaire was modified (Appendix S.2 in Supplementary Materials) and used for a consumer study in phase 6 where 106 men and women who were regular consumers of sweetpotato (Table S.3 in Supplementary Materials) were interviewed to identify their attitudes towards 7 sweetpotato genotypes. Due to poor internet connection, data were entered on printed ballots then transferred to Compusense.

Panelists rated overall liking, color liking and aroma liking of the samples on a 9-point hedonic scale ranging from 1 (dislike extremely) to 9 (like extremely). They also rated sweetness, mealiness and firmness on just-about-right scales ranging from 1 to 5 centered by just-about-right (JAR) at 3. These attributes were previously identified as the most important drivers for consumer preference of boiled or steamed sweetpotato in Uganda (Mwanga et al., 2021).

#### 2.4.2 Sample preparation and presentation for consumer sensory analysis

During the pilot study, samples for consumer sensory analysis were prepared according to the method outlined in section 2.2.4. However, to align with participatory plant breeding design, women identified from the local community prepared the sweetpotato in a culturally appropriate manner. Each woman was assigned a single variety. The women peeled the raw roots and placed them in water immediately to prevent browning, wrapped the roots in banana leaves and placed the bundle in saucepans matted with grass. They covered the wrapped sweetpotato roots with more banana leaves and added water to the saucepans to steam the sweetpotatoes over a three stone fireplace with burning wood. Roots were deemed ready when the loud bubbling sound of boiling water became quieter indicating reduced water levels. The sweetpotatoes were removed from the fire, unwrapped slightly and lightly pressed using fingers to confirm that they were cooked before serving.

In order to overcome the limitation of number of roots, roots of different sizes were cooked. Ready to eat sweetpotato roots were divided depending on size and medium sized roots were quartered while large roots were divided into 8 portions. The portions were wrapped in aluminum foil and labelled with random codes. Respondents were presented with the cooked samples monadically to evaluate in random order.

### 2.5 Data and statistical analysis

#### 2.5.1 Descriptive sensory analysis (Phase 1 to 6)

All sensory data were first organized in Microsoft Excel (Microsoft 365 Apps for enterprise, version 2102). For descriptive sensory analysis, data from genotypes served in duplicate (sensory replicates) were used to evaluate panel reliability in SPSS version 22 (IBM Corp. in Armonk, NY, 2013) by Fisher’s test with genotypes and panelists as fixed factors (Canul et al., 2011).

Data for descriptive sensory analysis at phase 3, phase 4, and phase 6 were analyzed in SPSS analysis of variance (ANOVA) models with genotypes as fixed variable and panelists as a random factor. To visualize relationships between the sensory attributes and the genotypes, principal component analysis (PCA) using mean scores for each replicate by the trained panel was run in XLSTAT (2020.5.1, Addinsoft, 2021) using the covariance option. Attributes that were not found to be significantly different among the varieties by ANOVA were excluded from the PCA. During phase 3, genotypes were not found to be different (p > 0.05) in caramel aroma, off -odor, floral flavor, bitter taste, and adhesiveness, hence the exclusion of these attributes from PCA. Attributes that were excluded from the PCA at phase 4 were: caramel aroma, off odor, floral flavor, bitter taste, fibrous appearance, cooked carrot flavor, crunchiness, and adhesiveness. Caramel aroma, off odour, degree of translucency, fibrous appearance, cooked carrot flavor, floral flavor, sweet taste, bitter taste, adhesiveness, and fibrousness were excluded at phase 6.

#### 2.5.2 Instrumental texture analysis (Phase 4 to 6)

With all texture data, means for each texture parameter, specifically average peak positive force and average positive area for the first and second compressions, were calculated. When analyzing data from descriptive sensory panel and instrumental texture analysis, a panel mean for each sensory attribute evaluated by the panel was calculated per genotype including those that were served in duplicate.

In phase 4, instrumental texture parameters were correlated with sensory firmness (descriptive sensory analysis) using Pearson correlation analysis. In phase 5, a linear regression model predicting sensory firmness was developed in XLSTAT with the best model procedure using 59 clones which had complete data from the DDBIO multi-location advanced field trial planted in 2021 as the training set and the 12 genotypes of the DDBIO advanced field trial from phase 4 as the validation set. To solve for multi-collinearity due to the strong correlation between instrumental texture parameters, only peak positive force (firmness) of the first compression was included in the model. Natural logarithms of the values of the predictors (dry matter and peak force) were entered in the model to ensure heteroscedasticity of the output residuals, and RMSE values of both the calibration and validation sets were calculated. The model was validated using descriptive sensory data from the panel and instrumental texture analysis of 39 genotypes from another population, MDP, of the same season.

#### 2.5.3 Penalty analysis with consumer acceptability tests (Phase 4 and phase 6)

To conduct penalty analysis, the 5 point just-about-right scale was collapsed into 3 categories by combining the lower end points (1 and 2), and upper point scales (4 and 5) (Ortega-Heras et al., 2019). Penalty analysis was conducted separately for each genotype using these categories and the overall liking data.

#### 2.5.4 Establishing proposed minimum and maximum values of instrumental texture parameters using consumer acceptability tests in on-farm trials (Phase 6)

Several measures from the firmness JAR question were plotted against instrumental texture measures to propose minimum and maximum values corresponding to consumer liking. Linear regression was applied to establish relationships between instrumental texture measurements of genotypes and frequencies of being ‘too soft’, ‘too hard’, or ‘just-about-right’ in firmness. Plots of the frequency of these responses were plotted against instrumental texture measures of firmness (peak force 1) and toughness (positive area 1) to produce linear prediction equations in Excel.

In order to select a suitable threshold, plots of overall liking and the frequency of responses to the just-about-right question on firmness were also plotted to identify the frequency associated with a minimum overall liking of 6, which corresponds to ‘like slightly’. These were used as cut-off frequencies to calculate proposed values of instrumental texture measures. The frequencies of respondents who found the sweetpotato samples ‘too soft’ or ‘just-about-right’ in firmness were entered in their respective prediction equation to calculate the values of texture firmness (peak force) and toughness (positive area). These output values were proposed as minimum values for instrumental texture parameters that would indicate sweetpotatoes of suitable firmness for Ugandan consumers.

## 3 Results

### 3.1 Lexicon for descriptive sensory analysis of steamed sweetpotato

#### 3.1.1 Initial lexicon draft and changes

The initial lexicon comprised 36 terms describing the aroma, appearance, flavor, and texture of steamed sweetpotato (Table S.4 in Supplementary Materials). The lexicon was reduced to the final 27 terms of which four described aroma and appearance while six described flavor and 13 described texture attributes. Several aroma terms were combined under the general category “off-odor”. Crumbliness was introduced in this iteration of the lexicon. Initially all texture terms were assessed in the mouth. However, during lexicon refinement, the panel indicated that attributes such as hardness, cohesiveness, crumbliness/mealiness and moisture release were more easily assessed by hand. Ugandans usually eat sweetpotato by hand.

#### 3.1.2 Reference products

The list of reference products, their preparation methods and associated attributes are shown in Table S.5 in Supplementary Materials. These items were all obtained from local produce markets and supermarkets and as such are readily available. They include farm produce, a wide variety of vegetables and legumes, teas, and common snacks which are consumed in Ugandan households.

#### 3.1.3 Final lexicon: definitions, scale anchors, and methods of assessment

The list of attributes in the final lexicon with definitions, scale anchors, and method of assessment are shown in Table 1. The definitions included here are not technical but rather simplified versions with local examples to make it easy for the panelists to understand.

**Table 1:** The final lexicon for evaluation of cooked sweetpotatoes by a trained descriptive sensory panel.

Assessment method	Descriptors	Simplified definition	Scale range
Aroma			
Once sample is received, slightly unwrap it, observe aroma with a single short whiff, close the foil, and mark your scores on the aroma scales	Sweetpotato	Smell of cooked sweetpotato	0 = none to 10 = very strong
	Caramel	Smell of burnt sugar or molasses (*sukaali gulu*)	0 = none to 10 = very strong
	Pumpkin	Smell of cooked pumpkin	0 = none to 10 = very strong
	Off-odor	Unusual smells in sweetpotato including potato, boiled beans, amaranth, herbal, floral, and pungent/acidic/rotting sweetpotato	0 = none to 10 = very strong

Appearance			
Visually assess the outer surface of the sweetpotato for orange color intensity	Orange color intensity	Intensity of orange color across the surface of the sample	0 = white, 1 = cream, 3 = yellow, 5 = yellow orange, 8 = orange, 10 = deep orange
Observe the cross-sectional cut for uniformity of color, degree of translucency and fibrousness	Uniformity of color	Evenness of color distribution across sample surface	0 = highly variable to 10 = consistent throughout
	Translucency	Quality of an object to allow light to pass through it but does not allow images to be distinguished such as a slice of steamed cucumber	0 = 100% chalky/opaque to 10 = 100% translucent
	Fibrous appearance	Presence of visible strings within sample mass	0 = none to 10 = extremely fibrous

Flavor			
Take a portion of the sample and chew slowly to score the intensity of the flavors	Sweetpotato	Intensity of the flavor of cooked sweetpotato	0 = none to 10 = very strong
	Pumpkin	Intensity of the flavor of cooked pumpkin	0 = none to 10 = very strong
	Cooked carrot	Intensity of the flavor of cooked carrot	0 = none to 10 = very strong
	Floral	Intensity of the flavor of flowers	0 = none to 10 = very strong
Take a portion of the sample and chew slowly to score the intensity of the basic tastes that you observe	Sweet	Taste of sugar	0 = not at all sweet to 10 = extremely sweet
	Bitter	Taste of quinine, strong coffee, *katunkuma (Solanum anguivi)*, *nakati (Solanum aethiopicum)*	0 = not bitter to 10 = extremely bitter

Texture in mouth			
Take a portion of sample and bite using front teeth (incisors) and assess fracturability.	Fracturability	Ease with which sample breaks into distinct pieces when bitten between incisors	0 = easily deforms to 10 = easily fractures
Take another portion and bite using back teeth (molars) and assess hardness.	Hardness in mouth	Amount of force required to compress product between molars	0 = extremely soft to 10 = hard
While chewing (chew down), assess crunchiness and moisture in mass (3 chews).	Crunchiness	Production of low-pitched sound while chewing certain foods such as carrot, cucumber	0 = not crunchy to 10 = extremely crunchy
	Moisture (in the mass)	Amount of moisture present in sample mass	0 = dry to 10 = extremely moist
After chewing 3 times, place sample between tongue and palate and assess crumbliness in mouth, adhesiveness, fibrousness, and smoothness	Crumbliness in mouth	Extent of powder like particles in sample mass	0 = not mealy to 10 = extremely mealy
	Adhesiveness	Amount of sample that adheres to oral surfaces	0 = none to 10 = very high
	Fibrousness	Presence of string like structures in mouth after chewing	0 = none to 10 = very high
	Smoothness	Degree of absence of grainy particles in mass	0 = grainy to 10 = very smooth
Take a portion of sample and chew until prompt to swallow to assess the rate of breakdown.	Rate of breakdown	Number of chews required to masticate a sample until you can swallow it	0 = very slow to 10 = very fast

Texture by hand			
Press down the center of the sample to evaluate the force required to compress sample	Hardness by hand	Amount of force required to compress sample	0 = very soft, 5 = firm, 10 = very hard
Take a portion of sample and press between fore finger and thumb to assess moisture release.	Moisture release	Attribute of food products to release moisture when pressure is applied such as cooked cucumber and French beans	0 = none to 10 = extremely moist
Attempt to make a ball from the sample to evaluate cohesiveness (moldability).	Cohesiveness (moldability)	Ease with which a ball like shape can be molded from sample	0 = falls apart to 10 = moldable
Rub a portion of sample between fingers to evaluate mealiness.	Crumbliness (mealiness)	Ease with which sample breaks into small particles upon rubbing	0 = not mealy to 10 = extremely mealy

### 3.2 Sensory profiles of sweetpotato genotypes and performance of trained panel after virtual training and sample evaluation outside the laboratory (office setting)

**Fig. 1:**
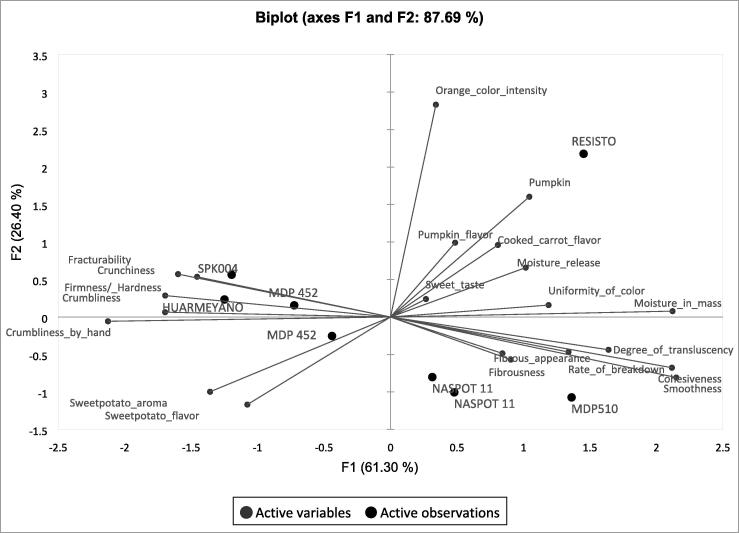
Principal component analysis (PCA) showing the correlation between sensory attributes of 6 sweetpotato genotypes (2 evaluated in duplicate) as evaluated by a trained sensory panel in office setting.

Generally, the results from the trained panel suggested that there was variation in the sensory profiles of the genotypes evaluated. Fig. 1 shows the PCA map drawn with 2 main components: F1 and F2 explaining 61 % and 26 % of the total variance among samples using 21 attributes. The first component separated sweetpotato genotypes with more moisture and cohesiveness on the right side of the plot (Resisto and MDP 510) from those that were crumblier on the left (Huarmeyano and SPK004). This component separated samples mostly based on texture. The second component separated the genotypes with high color intensity, particularly Resisto at the top of the plot from the white fleshed genotypes such as MDP 452, MDP 510 and NASPOT 11 located in the bottom half. After running a PCA with only sensory texture attributes, a plot of the first and third main components explaining 89 % of total variance (Figure S.3 in Supplementary Materials), revealed that MDP 510 was distinctly fibrous.

The replicates of NASPOT 11 and MDP 452 are located closely on the map in Fig. 1 indicating good panel consistency. There was no difference between replicates of these genotypes (p > 0.05) except that the mean score for smoothness among the replicates of MDP 452 was significant (mean and standard deviation 4 ± 2 replicate 1 vs 6 ± 1 replicate 2) (Table S.6 in Supplementary Materials).

### 3.3 Sensory profiles of 12 genotypes from the DDBIO advanced field trial planted in 2020

**Fig. 2:**
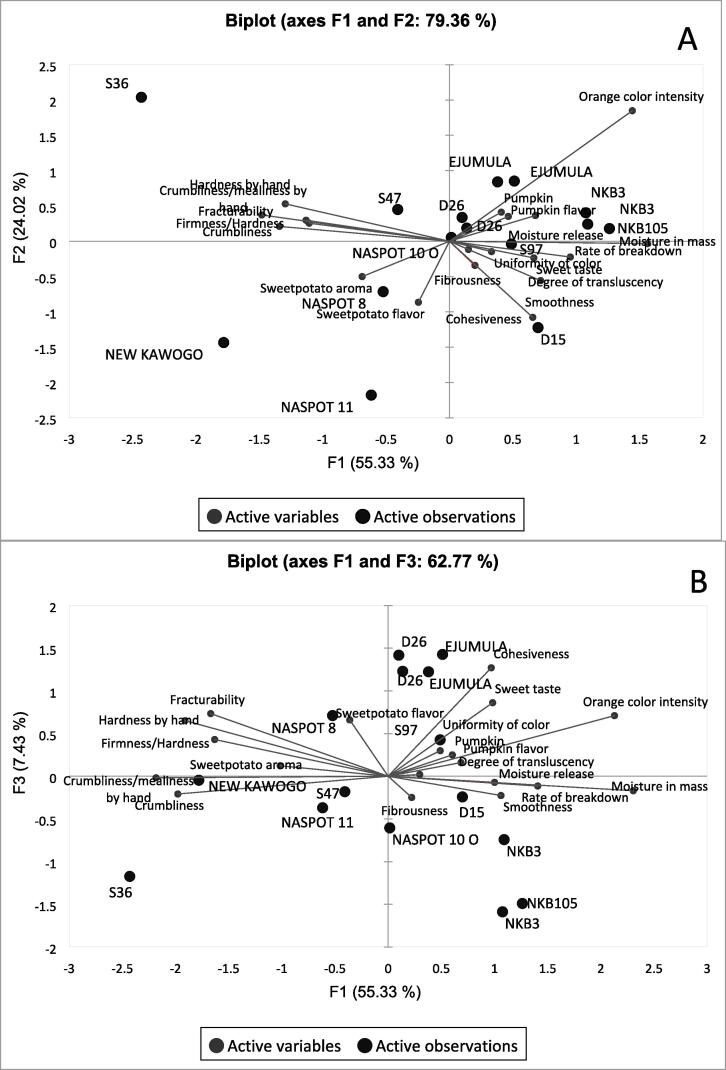
Principal component analysis maps showing the relationship between sensory attributes of 12 genotypes in advanced trial (with 3 evaluated in duplicate) as evaluated by a trained descriptive sensory analysis panel with A showing second principal component, F2 versus first principal component, F1 and B showing third principal component, F3 versus first principal component, F1.

The PCA of the 12 genotypes from the advanced trial are presented in Fig. 2. The plot shows the first three main components explaining 87 % of the variation among the genotypes according to 19 sensory attributes. Most texture attributes loaded on the first component.

The first component, F1, separated genotypes by texture attributes with firm and crumbly genotypes on the left (S36, New Kawogo) and soft and moist genotypes on the right (NKB3, NKB105). S36, an orange-fleshed genotype, was particularly firm while NKB3 and NKB105 were moist and soft. The second component, F2, separated varieties by orange color intensity and sweetpotato flavor. Deeply orange genotypes such as Ejumula, S36, NKB3 and NKB105 appeared towards the top of the plot, and NASPOT 11 and New Kawogo which are white, and cream fleshed towards the lower side of Fig. 2. NASPOT 10 O and D26 did not have significant loadings on the first two components (A) and had higher loadings with the third component (B).

**Fig. 3:**
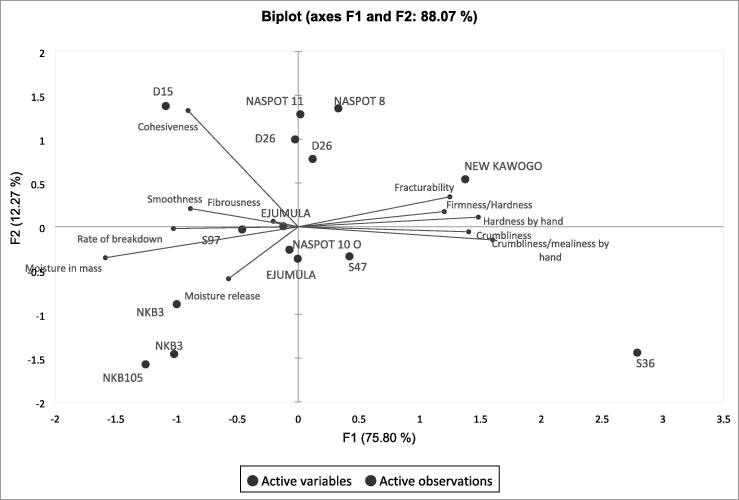
Principal component analysis map showing the relationship between sensory texture attributes of 12 genotypes in advanced trial (with 3 evaluated in duplicate) as evaluated by a trained descriptive sensory analysis panel.

Fig. 3 shows a PCA map with only sensory texture attributes. The two main components explain 88% of total variation. F1 separates moist genotypes (D15, NKB105, NKB3) on the left from S36 and New Kawogo on the right, which are mealy and firm. F2 separated genotypes by cohesiveness with highly cohesive genotypes (D26, NASPOT 11, NASPOT 8, and D15) appearing on the top half of the plot and the less cohesive genotypes (S36,NKB3 and NKB105) being in the bottom half.

#### 3.3.1 Correlation between sensory firmness and instrumental texture analysis of 12 sweetpotato genotypes from DDBIO advanced field trial planted in 2020

The dry matter and instrumental texture parameters for the 12 genotypes in the advanced trial are shown in Table S.7 in Supplementary Materials. The dry matter ranged from 28 % (NKB3) to 38 % (NASPOT 11). S36 and D26 had the highest peak force (firmness) and positive area (toughness), while NKB3 and NKB105 had the lowest.

There was good correlation between sensory firmness and parameters of instrumental texture especially peak positive force (firmness) and positive area (toughness) of the first curve (Table 2). Peak positive force was positively correlated with sensory firmness in mouth (r = 0.695) and sensory hardness by hand (r = 0.648). The correlation coefficients between positive area and sensory firmness in mouth (r = 0.748) and sensory hardness by hand (r = 0.715) were higher than those with peak positive force indicating a slightly stronger relationship. There was no correlation between dry matter and sensory firmness in this study.

**Table 2:** Correlation between sensory hardness, dry matter and various parameters of instrumental texture using 12 genotypes from DDBIO advanced field trial planted in 2020.

Variables	Hardness by hand	Firmness in mouth	Dry matter	Peak positive force 1	Positive force 2	Positive area 1	Positive area 2
Hardness by hand	1						
Firmness in mouth	**0.977**	1					
Dry matter	0.236	0.334	1				
Peak positive force 1	**0.695**	**0.648**	0.182	1			
Peak positive force 2	0.459	0.402	0.050	**0.897**	1		
Positive area 1	**0.748**	**0.715**	0.324	**0.935**	**0.787**	1	
Positive area 2	0.498	0.456	0.080	**0.920**	**0.877**	**0.757**	1
Values in bold are significantly different from 0p < 0.05							

### 3.4 Linear regression model describing the relationship between sensory firmness and instrumental texture parameters

**Fig. 4:**
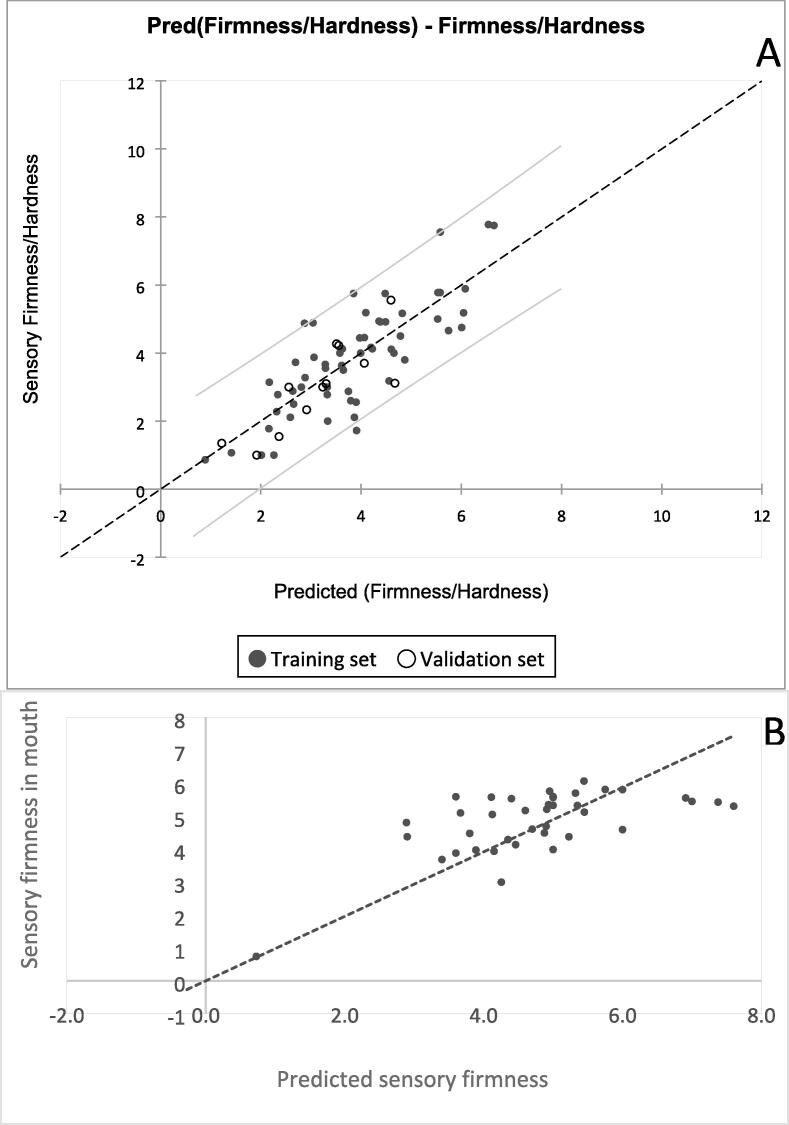
Plots of (A) sensory firmness versus predicted sensory firmness from the developed multiple linear regression model using material from DDBIO population and (B) sensory firmness versus predicted sensory firmness from the developed linear regression model using MDP population.

A multiple linear regression model (S.1 in Supplementary Materials) was developed to explain variation of sensory firmness in mouth among sweetpotato genotypes by dry matter and peak positive force of the first compression. Fig. 4 shows a plot of sensory firmness versus predicted firmness (A) of the selected model. The model explained 65 % variation for the calibration set and 67 % variation in the validation set. The RMSE values for the calibration and validation sets were 0.9 and 0.7, respectively. A plot of sensory firmness versus predicted sensory firmness from the model when validated using results from an additional 39 genotypes is shown in Fig. 4 panel B. In this case, the RMSE was 1.0. indicating that the model could predict the sensory firmness with an accuracy of plus or minus one unit on the 11-point intensity scale used by the trained panel.

### 3.5 Sensory profiles of 7 genotypes evaluated in on-farm trials during phase 6

**Fig. 5:**
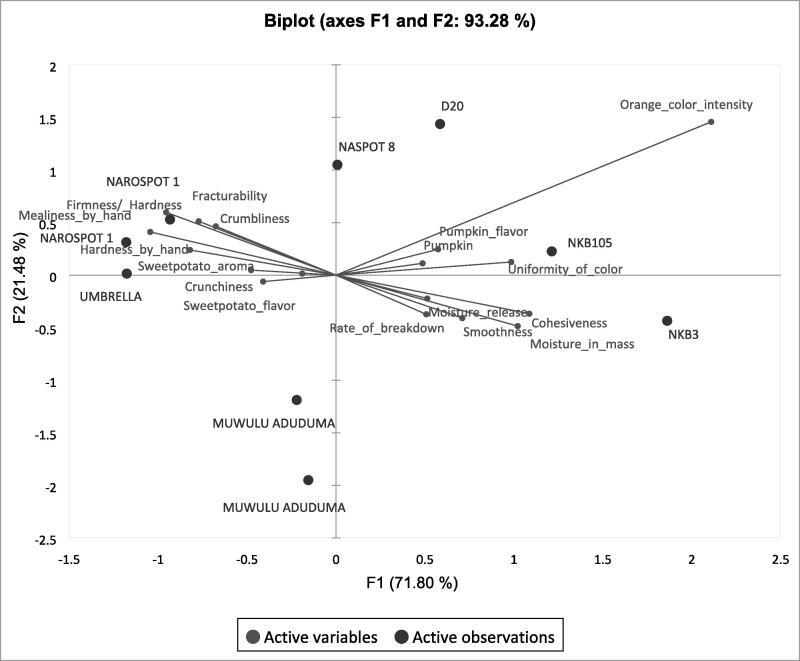
Principal Component Analysis showing the relationship between sensory attributes of 7 genotypes (2 served in duplicate) used in on-farm trails.

The sensory profiles developed by the trained panel of the 7 genotypes evaluated in on-farm trials are shown on the PCA, which explains 93 % of the total variation among the genotypes (Fig. 5). Orange fleshed genotypes (D20, NASPOT 8, NKB3 and NKB105) appear on the right of the first component, while white, cream and yellow fleshed genotypes (NAROSPOT 1, Umbrella and Muwulu Aduduma) are on the left. Umbrella and the replicates of NAROSPOT 1 were also firm and crumbly compared to NKB3 which was moist. Consistent with observations from previous phases, sweetpotatoes with pumpkin aromas and flavors appeared separately from those with higher intensities of sweetpotato aroma and flavors were on the map.

### 3.6 Overall liking and penalty analysis of 7 genotypes evaluated in on-farm trials

The mean overall liking for the different sweetpotato genotypes ranged from 5 (NKB3) to 7 (D20, NASPOT 8, NAROSPOT 1 and Umbrella) (Table 3). Using the threshold of a 2 unit penalty value (mean drop = 2) and 30 % minimum just-about-right response frequency, all the genotypes were penalized for not being mealy enough. Only NKB3 and NKB105 were penalized for not being sweet enough. In regards to firmness, only D20, NAROSPOT 1 and Umbrella were considered to be firm enough.

**Table 3:** Penalty (mean drop) and corresponding respondent frequencies from 5-point just-about-right (JAR) questions, and mean overall liking rating (9-point hedonic scale) of seven genotypes evaluated by a consumer panel (n = 106) in on-farm trials.

Genotype	JAR score^1^				Overall liking^2^
		Overall liking penalty (frequency responses, % n)			
		Sweet taste	Firmness	Mealiness	mean ± SD
D20	JAR score < 3	2 (24 %)	2 (24 %)	2 (32 %)	7 ± 2 ^a^
	JAR score > 3	2 (10 %)	1 (9 %)	1 (4 %)	
MUWULU ADUDUMA	JAR score < 3	3 (24 %)	3 (49 %)	2 (58 %)	6 ± 2 ^bc^
	JAR score > 3	1 (12 %)	3 (3 %)	0 (1%)	
NAROSPOT 1	JAR score < 3	3 (30 %)	2 (22 %)	2 (30 %)	7 ± 2 ^ab^
	JAR score > 3	2 (4.7 %)	1 (7 %)	0 (5 %)	
NASPOT 8	JAR score < 3	2 (29 %)	2 (34 %)	2 (38 %)	7 ± 2 ^ab^
	JAR score > 3	1 (11 %)	2 (6 %)	−1 (2 %)	
NKB105	JAR score < 3	3 (41 %)	2 (48 %)	2 (55 %)	6 ± 3^c^
	JAR score > 3	0.7 (8.5 %)	2 (4 %)	5 (1 %)	
NKB3	JAR score < 3	2 (48 %)	3 (83 %)	2 (82 %)	5 ± 3 ^d^
	JAR score > 3	0.1 (13 %)	2 (1 %)	0 (0 %)	
UMBRELLA	JAR score < 3	3 (20 %)	3 (27 %)	2 (38 %)	7 ± 3 ^ab^
	JAR score > 3	2 (12 %)	3 (5 %)	−1 (3 %)	

1 JAR rated on a 5 point just-about-right scale where 3 = just-about-right, JAR < 3 = too little, and JAR > 3 = too much.

2 Overall liking rated on a scale ranging from 1 (dislike extremely) to 9 (like extremely). Data analyzed by ANOVA with means separation by Duncan’s Multiple Range test; values in this column with different superscript lowercase letters are significantly different (P < 0.05).

### 3.7 Proposed minimum levels for instrumental texture parameters

**Fig. 6:**
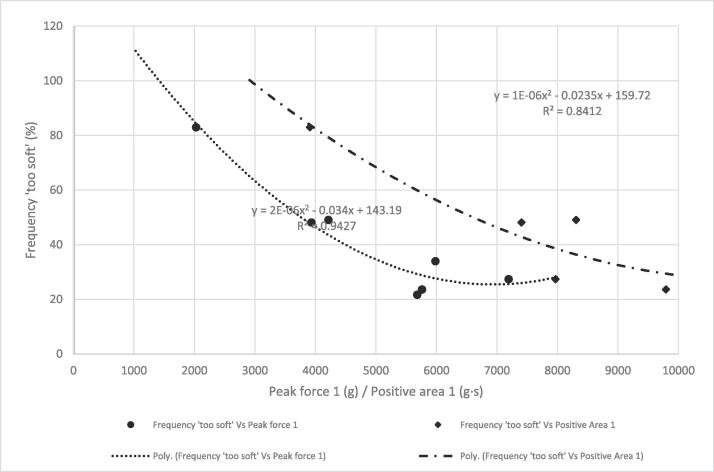
Frequency plot showing proportion of respondents who perceived sweetpotato samples in on-farm trials to be ‘too soft’ versus peak force 1 and positive area under curve.

**Fig. 7:**
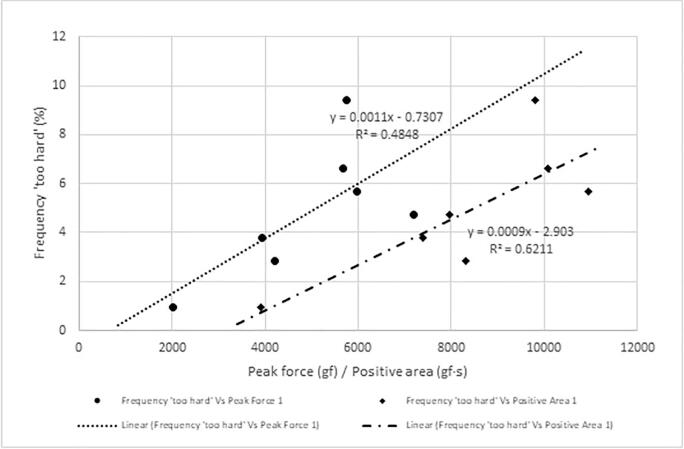
Frequency plot showing proportion of respondents who perceived sweetpotato samples in on-farm trials as being 'too hard' versus instrumental texture parameters of peak positive force 1 (gf) and positive area 1 (gf·s).

**Fig. 8:**
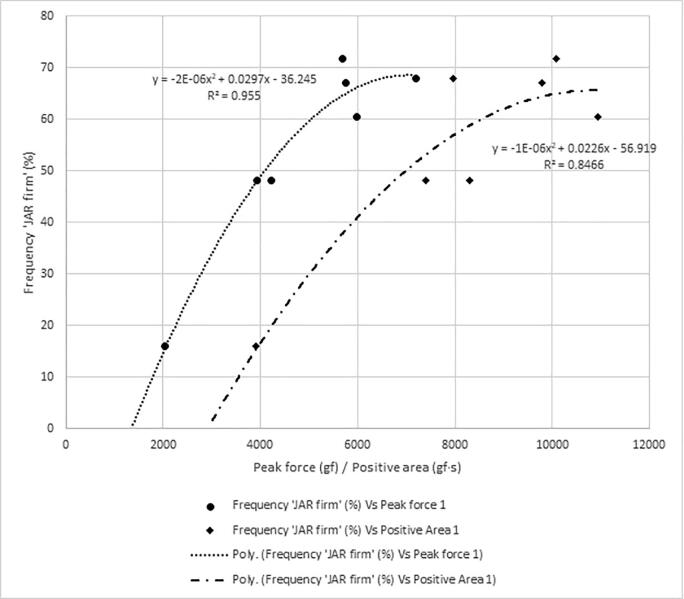
Frequency plot showing proportion of respondents who perceived the firmness of sweetpotato samples from on-farm trials in Hoima to be ‘just-about-right’ versus instrumental texture parameters of peak positive force 1 (gf) or positive area 1 (gf·s).

**Fig. 9:**
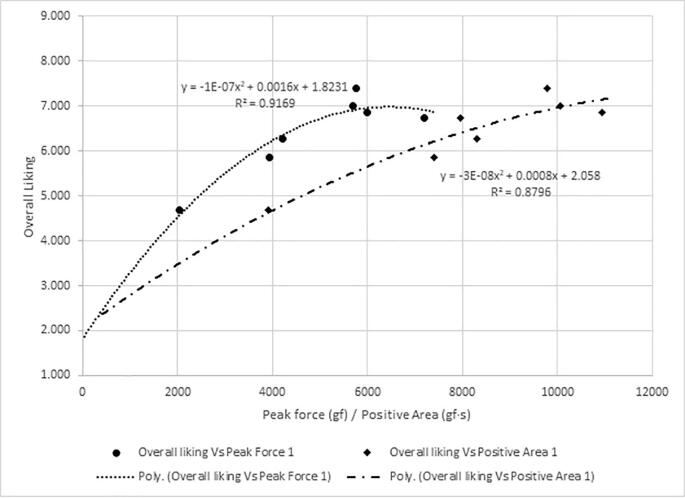
Plot showing average overall liking for sweetpotato samples rated by consumers in on-farm trials in Hoima verses instrumental texture parameter of peak positive force 1 (gf) and positive area (gf·s).

Fig. 6, Fig. 7, Fig. 8 show the frequency plots for responses ‘too soft’, ‘too hard’ and ‘just-about-right’, respectively, in relation to instrumental texture measurements and the equation of the trendlines. The percentage of respondents who perceived any of the seven samples to be’too soft’ (Table 3) was wide (22 % to 83%) compared to those who perceived any sample to be ‘too hard’ (0 % to 9 %). The associations between the instrumentally measured firmness (peak force) and the percentage of consumers that found the sweetpotatoes ‘too soft’ or ‘just-about-right’ in firmness were strong (R^2^ = 0.96 and R^2^ = 0.85 respectively). Similarly, there was a strong positive relationship between instrumental firmness and overall liking of the samples (Fig. 9, R^2^ = 0.92), whereas the relationship between instrumentally measured firmness and respondents who found any sample ‘too hard’ was weak (R^2^ = 0.48). Due to the low frequency of samples perceived as ‘too hard’, it was not considered for establishing maximum levels for instrumental texture firmness.

Figure S.4 shows the relationship between overall liking and responses to just-about-right question on firmness. Using these relationships, cut-off values for frequency of respondents perceiving samples to be ‘too soft’, ‘just-about-right’ firm, and ‘too hard’ corresponding with a minimum overall liking of 6 were 50 %, 46 % and 4 %, respectively. Using the prediction models, 50 % response of ‘too soft’ corresponded with an average peak force of 3435 gf (∼3400 gf) and positive area of 6426 gf.s (∼6400 gf·s). About 46 % consumers would perceive sweetpotato firmness to be ‘just-about-right’ when peak force is 3682 gf (∼3700 gf) and positive area is 6323 gf·s (∼6300 gf·s). Thus, the minimum values of peak force and positive area were set at 3700 gf and 6400 gf·s, respectively for large scale screening of sweetpotato genotypes for consumer preferred firmness.

Even though the relationship between the proportion of consumers who perceived sweetpotato as being too firm and instrumental texture parameters was linear, the small range of responses made it difficult to determine the relationship at higher proportions. It was therefore difficult to make conclusions on the maximum values of instrumental texture parameters in the current study.

## 4 Discussion

In the early stages of lexicon development, panelists used a Luganda term, “kiwutta” to describe the texture, taste, and flavor of some sweetpotatoes. Upon further discussions with the panel, it was identified that “kiwutta” referred to poor quality characterized by translucent appearance, extreme hardness, moisture release, crunchiness, and high sweetness and intense floral flavors. Its derivative term muwutta was used to refer to a glassy texture by consumers of boiled potato (Mudege et al., 2021) observed when potato does not go through glass transition upon heat treatment (boiling) thus maintaining the uncooked hard texture associated with its glassy state. Another Luganda hedonic term, “kukumuuka” was associated with sweetpotato that was not translucent and whose texture was dry and powdery akin to crumbly/mealy texture like the meaning of the term as used by potato consumers reported by Mudege and colleagues.

There were two iterations of lexicon developed for steamed sweetpotato with the second refining and reducing the number of descriptive terms. Comprehensive sensory characterization of sweetpotato using many descriptors is ideal but would be challenging for routine use with breeding trials because there are many samples to analyze. Fewer attributes reduce response burden on the panelist and could thus also contribute to improved panel performance (ISO, 2005). Since there was no significant variation among sweetpotato genotypes regarding caramel aroma, off odor, floral flavor, bitter taste, adhesiveness, fibrous appearance, and cooked carrot flavor, the exclusion of these attributes from routine analysis could be considered, especially when the objective is to differentiate between genotypes in advanced stages of breeding such as the ones used for the current study. It is possible that important negative characteristics such as fibrousness, bitter taste and off-flavor are effectively screened against earlier in the breeding pipeline. However, this may only be limited to CIP’s sweetpotato breeding strategy and there is need for contextual consideration when deciding to adapt this strategy. Another approach would be to include either one of the attributes that appear closely related according to the PCA (Fig. 1 and Fig. 2) such as hardness by hand or firmness/hardness in mouth, crumbliness by hand or crumbliness in mouth, fracturability or firmness/hardness in mouth. However, the close association between such sensory attributes in sweetpotato need to be confirmed by studies which include more genotypes.

The lexicon was modified progressively as panelists were trained with exposure to sensory characteristics of different sweetpotato genotypes, like the concept of a ‘living lexicon’ as shown by Chambers et al. (2016) for coffee. Although, the genotypes used for this lexicon may not cover the entire product space of sweetpotato (for example, no purple fleshed sweetpotatoes were included), the selection was sufficient for our study in Uganda since genotypes outside this range are currently rare. Therefore, this lexicon can be used as the basis for sensory profiling of white, cream, yellow, and orange-fleshed sweetpotatoes. It can be modified for use with purple sweetpotato and closely related commodities in contexts different from that of the current study.

The lexicon shares common terms with others such as the lexicon for boiled sweetpotato by Leighton et al. (2010) with some slight variations in the nomenclature. Texture attributes such as firmness, fibrousness, cohesiveness, and moistness are common to those identified for fried sweetpotato (Sato et al., 2018, Dery et al., 2021) and baked sweetpotato (Leksrisompong et al, 2012), demonstrating a degree of similarity in sweetpotato across agronomic environments and preparation methods. The methods of assessment or references were modified further to suit evaluation of steamed sweetpotato and include items familiar to the panelists’ backgrounds such as local food products and vegetables. While assessing the sensory quality of potato, Bough, Holm and Jayanty (2019) observed more variation between genotypes than preparation method and this may be the reason why the lexicons have similar terms. This implies that our lexicon is quite versatile and could be adapted for use in other regions and for other sweetpotato products, which is especially important due to the ongoing diversification of the sweetpotato product range in sub-Saharan Africa, including increased promotion of puree and puree-based products. There is potential for this lexicon to be used to evaluate sweetpotato genotypes and determine their suitability for various uses. Moreover, we include reference products in our definitions which are easy to interpret in case of use with another panel even if it is in a different location (Suwonsichon, 2019). This sweetpotato lexicon includes terms that have also been used to describe other roots and tuber crops such as dessert bananas (Buguad et al., 2011) due to the similarity in structure and composition of starch and other carbohydrates of these foods which determine their texture and taste (Joanna et al., 2019).

The sensory panel was able to discriminate samples after virtual training and evaluation of samples outside the laboratory when prepared in a centralized cooking area. The different genotypes (Huarmeyano, SPK004, Resisto, MDP 452 and MDP 510) were differentiated based on their overall sensory profiles, and the duplicates of NASPOT 11 and MDP 452 were close together indicating the good panel performance in this setting. Nonetheless, the validity of conducting descriptive sensory analysis outside the lab cannot be concluded from the current study since panel performance was not compared with the conventional method. Future studies seeking to recommend alternative spaces in which to conduct descriptive sensory analysis should compare with laboratory evaluations.

One important way of integrating descriptive sensory analysis with breeding is by developing rapid instrumental methods that can help in screening potential genotypes (Bough et al., 2019). The peak positive force and positive area of the first cycle of the TPA method for evaluating instrumental texture showed positive linear correlation with sensory firmness in this study. A previously established robust method for evaluating firmness of boiled sweetpotato using a wedge fracture test correlated sweetpotato texture with optimal cooking time (Banda et al., 2021a). In that study, the compression test did not discriminate between sweetpotato genotypes. In contrast, by applying a modified version of the compression test and sample preparation method, the current study found that the peak positive force and positive area of the first compression were useful for discriminating sweetpotato genotypes by their instrumental firmness.

The linear regression model developed to predict sensory firmness using instrumental texture consistently confirmed that there was a good relationship between the two measures of sweetpotato firmness. The model had good precision but only explained 65 % of total variation among genotypes by sensory texture. Nonetheless, with this prediction the instrumental texture method developed in this study will still be useful in helping breeders to objectively screen sweetpotato genotypes by sensory firmness earlier in the breeding process.

It is possible that there are other instrumental texture or biochemical measures that could explain sensory firmness which could be added to the model and increase the variation explained and improve prediction of the model. The current method was correlated with sensory firmness and future studies should develop alternative methods that could predict textural aspects of sweetpotato related to other human perceptions of sweetpotato texture such as mealiness and fibrousness. Measures that investigate the visco-elastic properties of the sweetpotato such as stress-relaxation could be useful. Instrumental texture analysis such as the method developed in this study is simple and practical for breeding programs which could be a limitation of other texture measures. The established instrumental texture analysis method should be used to complement but not replace sensory profiling by trained descriptive sensory panels in breeding programs. Its application would be particularly useful in earlier stages of breeding when there are many genotypes to screen. It may still be necessary to use descriptive sensory panels to profile genotypes later in the breeding cycle when there are fewer genotypes and enough roots.

According to the consumer evaluation and penalty analysis, D20, NASPOT 8, NAROSPOT 1 and Umbrella were the most preferred varieties and NKB3 was the least preferred variety. NASPOT 8 has previously been indicated as one of the most preferred varieties based on sensory quality (Mwanga et al., 2021). D20, NKB105 and NKB3 were test clones in the current study and results showed that D20, an orange fleshed variety was well liked by consumers, comparing well with the leading market varieties and one local variety (Umbrella) while performing better than another local variety (Muwulu Aduduma).

In addition to validating an instrumental method for evaluating firmness of steamed sweetpotato, the study proposes a minimum (3700 gf) average positive peak force of the first cycle for use as a selection criterion when screening sweetpotato genotypes in breeding trials for consumer acceptance. Following this criterion, among the 12 genotypes of the DDBIO advanced field trial planted in 2020, only three test clones: D26, S36 and S47 would qualify to proceed to the next breeding stage while NKB3 and NKB105 could have been excluded from the on-farm trial that followed. Use of this proposed minimum value of peak force screening criteria proposed in this study requires validation in a study with genotypes of a wider range of firmness, especially firmer genotypes.

### 4.1 Limitations of the study

Breeding trials typically produce few roots which limits the availability of sample material for the various quality analyses conducted to inform screening of sweetpotato genotypes. Consumer testing of food products typically requires each consumer to be presented with similar size and shape of the product. Here roots of varying sizes and shapes were cooked, and the medium and large roots were divided differently in order to serve the large number of consumers. Consumer acceptability tests were also conducted with residents of one community and the preferred ranges of sweetpotato firmness could vary by region. Nonetheless, the study establishes protocols for efficient breeding based on consumer preferred texture parameters. Further work is required to develop screening criteria for appearance and flavor characteristics.

## 5 Conclusion

This study established a trained descriptive sensory analysis panel for sweetpotato in Uganda. A complete sensory lexicon, preparation and evaluation protocol for sweetpotato evaluation was developed. A method for instrumental texture analysis of steamed sweetpotato using TPA was validated and used to establish the lower critical value for consumer liking of sweetpotato firmness. This criterion can be used by breeders to efficiently select potential genotypes from the large sets of genotypes in the breeding pipeline.

## CRediT authorship contribution statement

**Mariam Nakitto:** Conceptualization, Methodology, Validation, Formal analysis, Investigation, Data curation, Writing – original draft, Writing – review & editing, Visualization. **Suzanne D. Johanningsmeier:** Methodology, Formal analysis, Investigation, Writing – original draft, Writing – review & editing. **Mukani Moyo:** Methodology, Investigation, Writing – original draft, Writing – review & editing, Supervision. **Christophe Bugaud:** Methodology, Validation, Investigation, Writing – review & editing, Supervision. **Henriette de Kock:** Methodology, Writing – review & editing, Visualization, Supervision. **Layal Dahdouh:** Methodology, Validation, Formal analysis, Writing – review & editing, Supervision. **Nelly Forestier-Chiron:** Methodology, Investigation, Validation, Writing – review & editing. **Julien Ricci:** Investigation, Validation, Writing – review & editing. **Elizabeth Khakasa:** Investigation, Writing – review & editing. **Reuben T. Ssali:** Methodology, Resources, Writing – review & editing, Project administration. **Christian Mestres:** Methodology, Formal analysis, Writing – review & editing, Supervision, Project administration. **Tawanda Muzhingi:** Conceptualization, Methodology, Writing – original draft, Supervision, Project administration.

## Declaration of Competing Interest

The authors declare that they have no known competing financial interests or personal relationships that could have appeared to influence the work reported in this paper.
