# Supplementary material for: Sensory guided selection criteria for breeding consumer-preferred sweetpotatoes in Uganda
Source: Food Qual Prefer. 2022 Oct;101:104628. doi: 10.1016/j.foodqual.2022.104628 (PMC9247747; doi:10.1016/j.foodqual.2022.104628)
Supplement: Supplementary data 1 [file mmc1.docx]

**Supplementary Tables**

**Table S.1.** Characteristics and sources of sweetpotato genotypes used in different phases of the study

| **Activity/day** | **Genotype** | **Flesh color** | **Source** |
| --- | --- | --- | --- |
| Phase 1 : Lexicon development (DSA) | | | |
| Day 1 | Dimbuka Bukalula | Cream | CIP, Namulonge |
|  | NASPOT 1 | Cream | CIP, Namulonge |
|  | NASPOT 8 | Yellow-orange | CIP, Namulonge |
|  | NASPOT 11 | Cream | CIP, Namulonge |
|  | Resisto | Deep orange | CIP, Namulonge |
|  | Dimbuka-Bukalula, watery | Cream | CIP, Namulonge |
| Day 3 | Local landrace, no name | Yellow | Makerere South market |
|  | Local landrace, no name | White | Makerere South market |
| Day 4 | Ejumula | Orange | CIP, Namulonge |
|  | NASPOT 8 | Yellow-orange | CIP, Namulonge |
|  | NASPOT 11 | Cream | CIP, Namulonge |
| Phase 2: Initial panel training sessions (DSA) | | | |
| Day 1 | Huarmeyano^1^ | Yellow | CIP, Namulonge |
|  | Resisto | Deep orange | CIP, Namulonge |
|  | Magabali | Cream | CIP, Namulonge |
| Day 2 | NASPOT 7^1^ | Orange | CIP, Namulonge |
|  | NASPOT 10 O | Orange | CIP, Namulonge |
|  | Mugande | Cream | CIP, Namulonge |
| Day 3 | NAROSPOT 1^1^ | Cream | Farmer, Kalagi |
|  | Kyadodondo^1^ | White | Farmer, Kalagi |
|  | | | |
| Phase 3: Virtual panel training during COVID-19 pandemic and sample evaluation in office settings (DSA) | | | |
| Day 1 | SPK004^2^ | Yellow-orange | CIP, Kabale |
|  | NASPOT 11^1^ | Cream | CIP, Kabale |
|  | Resisto | Deep orange | CIP, Kabale |
| Day 2 | MDP 452^1^ | Yellow | CIP, Kabale |
|  | Huarmeyano | Yellow | CIP, Kabale |
|  | MDP 510 | Cream | CIP, Kabale |
|  | | | |
| Phase 4 : Exploring relationship between sensory texture and instrumental texture parameters and pilot consumer study (DSA, instrumental texture analysis, consumer acceptability tests) | | | |
| Day 1 | D26 ^1^ | Orange | NARO, Rwebitaba |
|  | D15 | Orange | NARO, Rwebitaba |
|  | New Kawogo | White | NARO, Rwebitaba |
|  | NASPOT 8 ^3^ | Yellow-orange | NARO, Rwebitaba |
|  | Ejumula ^1^ | Orange | NARO, Rwebitaba |
|  | NASPOT 10 O ^3^ | Orange | NARO, Rwebitaba |
| Day 2 | NASPOT 11 ^3^ | Cream | NARO, Rwebitaba |
|  | NKB 3 ^1,3^ | Orange | NARO, Rwebitaba |
|  | S47 ^3^ | Orange | NARO, Rwebitaba |
|  | S97 | Orange | NARO, Rwebitaba |
|  | NKB105 | Orange | NARO, Rwebitaba |
|  | S36 | Orange | NARO, Rwebitaba |
| Phase 5 : Developing relationship between sensory firmness and instrumental firmness (DSA, instrumental texture analysis) | | | |
|  | 1.44 | Orange | NARO, several locations ^4,5^ |
|  | D11 | Orange | NARO, several locations ^4,5^ |
|  | D15 | Orange | NARO, several locations ^4,5^ |
|  | D20 | Orange | NARO, several locations ^4,5^ |
|  | D26 | Orange | NARO, several locations ^4,5^ |
|  | NKB3 | Orange | NARO, several locations ^4,5^ |
|  | NKB105 | Orange | NARO, several locations ^4,5^ |
|  | S36 | Orange | NARO, several locations ^4,5^ |
|  | S47 | Orange | NARO, several locations ^4,5^ |
|  | S97 | Orange | NARO, several locations ^4,5^ |
|  | Ejumula | Orange | NARO, several locations ^4,6^ |
|  | New Kawogo | White | NARO, several locations ^4,6^ |
|  | NASPOT 8 | Yellow-orange | NARO, several locations ^4,6^ |
|  | NASPOT 10 O | Orange | NARO, several locations ^4,6^ |
|  | NASPOT 11 | Cream | NARO, several locations ^4,6^ |
| Phase 6 : Sensory and instrumental texture analysis of on-farm trials in Hoima (DSA, instrumental texture analysis, consumer acceptability tests) | | | |
|  | D20 ^3^ | Orange | NARO, Hoima on-farm trial ^5^ |
|  | Muwulu Aduduma ^1,3^ | White | NARO, Hoima on-farm trial ^7^ |
|  | NAROSPOT 1 ^1,3^ | Yellow | NARO, Hoima on-farm trial ^6^ |
|  | NASPOT 8 ^3^ | Yellow-orange | NARO, Hoima on-farm trial ^6^ |
|  | NKB3 ^3^ | Orange | NARO, Hoima on-farm trial ^5^ |
|  | NKB105 ^3^ | Orange | NARO, Hoima on-farm trial ^5^ |
|  | Umbrella ^3^ | Yellow | NARO, Hoima on-farm trial ^7^ |
| The analyses conducted at each phase are indicated in parentheses  DSA = Descriptive sensory analysis conducted by the trained panel  CIP = International Potato Center  NARO = National Agricultural Research Organisation  ^1^ Genotypes served to trained panel in duplicate  ^2^ Was sprouting  ^3^ Genotypes evaluated for consumer acceptability by untrained respondents  ^4^  Clones from Namulonge : 1.44, D11, D15, D20, D26, NKB3, S36, S47, S97, Ejumula, New Kawogo, NASPOT 8, NASPOT 10 O, NASPOT 11; Clones from Arua : D11, D15, D20, D26, NKB3, S36, S97, Ejumula, NASPOT 8, NASPOT 10 O, NASPOT 11; Clones from Serere : D11, D15, D20, D26, NKB3, NKB 105, S36, S97, Ejumula, NASPOT 8, NASPOT 10 O; Clones from Bulindi : 1.44, D11, D20, D26, NKB3, S36, S97, Ejumula, NASPOT 8, NASPOT 10 O, NASPOT 11; Clones from Rwebitaba : 1.44, D11, D15, D20, D26, NKB3, NKB105, S36, S97, Ejumula, New Kawogo, NASPOT 8, NASPOT 10 O, NASPOT 11  ^5^ Test: genotypes being studied for selection and potential release as new varieties  ^6^ Check: released or local varieties of known agronomic performance  ^7^ Local: landraces whose agronomic performance is unknown | | | |

**Table S.2.** Sociodemographic characteristics and sweetpotato consumption patterns of consumer respondents in pilot study (n=23)

| **Characteristic** | **Descriptive statistic** |
| --- | --- |
| 1. Sociodemographic characteristics | Mean ± SD |
| Age | 31 ± 9 |
|  |  |
| Sex | n (%) |
| Male | 13 (57) |
| Female | 10 (43) |
| Occupation |  |
| Formal | 9 (39) |
| Informal | 10 (44) |
| None | 4 (17) |
| 1. Sweetpotato consumption characteristics |  |
| When do you eat sweetpotato | n (%) |
| Breakfast | 3 (13) |
| Lunch | 18 (78) |
| Supper | 2 (9) |
| Frequency of sweetpotato consumption |  |
| Everyday | 3 (13) |
| Several times a week | 12 (52) |
| Once a week | 5 (22) |
| Several times a month | 1 (4) |
| Once a month | 2 (9) |

**Table S.3.** Sociodemographic and sweetpotato consumption characteristics of consumer respondents in on-farm trials (n = 106)

| Characteristic | Descriptive statistic |
| --- | --- |
| Sex | n (%) |
| Female | 78 (74) |
| Male | 28 (26) |
| Age category |  |
| 18 – 24 years old | 15 (14) |
| 25 – 34 years old | 21 (20) |
| 35 – 44 years old | 23 (22) |
| 45 – 54 years old | 19 (18) |
| 55 – 64 years old | 16 (15) |
| 65 years and older | 12 (11) |
| Type of occupation |  |
| Formal | 4 (4) |
| Informal | 97 (91) |
| None | 5 (5) |
| Frequency of sweetpotato consumption |  |
| Everyday | 13 (12) |
| Two to six days a week | 83 (78) |
| Two to three days a month | 10 (10) |
|  |  |

**Table S.4.** List of terms in initial and final lexicon for steamed sweetpotato

| **Category** | **Terms in initial lexicon** | **Terms in final lexicon** |
| --- | --- | --- |
| Aroma | Sweetpotato, caramel, pumpkin, yam, cooked banana leaves, boiled corn, posho (cornmeal), boiled ‘Irish’ potato1, boiled beans1, green/ amaranth1, herbal1, pungent/acidic/rotting sweetpotato1 | Sweetpotato, caramel, pumpkin, off-odor |
| Appearance | Orange color intensity, uniformity of color, degree of translucency, fibrous appearance | Orange color intensity, uniformity of color, translucency, fibrous appearance |
| Flavor | Sweetpotato, pumpkin, cooked carrot, floral, sweet taste, sour taste, bitter taste | Sweetpotato, pumpkin, cooked carrot, floral, sweet taste, sour taste, bitter taste |
| Texture in mouth | Surface roughness, springiness, fracturability2, firmness/hardness, crunchiness, moisture release/watery, moisture in mass, adhesiveness/stickiness, uniformity of texture, cohesiveness/moldability, fibrousness, smoothness, rate of breakdown | Fracturability, firmness/hardness, crunchiness, moisture in mass, adhesiveness/stickiness, fibrousness, smoothness, rate of breakdown |
| Texture by hand |  | Moisture release/watery, cohesiveness/moldability, crumbliness/mealiness |
| Italicized terms in initial lexicon were omitted in final lexicon  ^1^ Aroma terms in initial lexicon combined to make the term off odor in final lexicon  ^2^ The fracturability scale initially had crumbly on one end and highly fracturable on the other end. After revisions, the scale anchors for fracturabilty changed to range from easily deforms on the low end to easily fractures on the high end of the scale, and crumbliness was made an independent term in final lexicon | | |

**Table S.5.** List of reference food products, preparation method and associated attributes

| **Reference sample** | **Preparation method** | **Associated attributes** |
| --- | --- | --- |
| Eggs (market) | Cooked in boiling water for 10 minutes then cooled to room temperature in cold water | Yolk: smoothness, firmness (soft), crumbliness (high), moisture in mass (dry), rate of breakdown (fast)  Egg white: white, fracturable |
| Pumpkin (*Cucurbita* spp.) | Sliced, steamed for 38 minutes | Pumpkin aroma, pumpkin flavor, |
| French beans (*Phaseolus vulgaris*) | Steamed whole for 11 minutes | Moisture release, translucency, crunchiness, sweetness |
| Potato (*Solanum tuberosum*) | Steamed whole for 34 minutes | Boiled ‘Irish’ potato aroma |
| Carrot (*Daucus carota L.*) | Sliced then steamed for 28 minutes | Cooked carrot aroma and flavor |
| Amaranth (*Amaranthus* spp.) | Steamed for 10 minutes | Green/amaranth aroma, |
| Taro aka ‘yam’ (*Diascorea esculenta)* | Peeled, divided into small portions, and steamed for 26 minutes | Yam aroma |
| Common bean (*Phaseolus vulgaris*) | Boiled for 25 minutes | Boiled bean aroma |
| Cassava dough | Cassava flour added to boiling water and stirred to make a thick paste | Adhesiveness/stickiness |
| Cucumber (*Cucumis sativus*) |  |  |
| Riham ginger biscuits (Hariss International Limited, Kampala, Uganda) |  | Smoothness (grainy), Surface roughness (one side rough, other side smooth) |
| Kericho Gold Green tea and Jasmine |  | Floral aroma |
| Heath and Heather Chamomile tea |  | herbal |
| Pringles Original Potato Crisps (Kellogg’s, Battle Creek, MI, USA) |  | Uniformity of texture, Uniformity of color, surface roughness, crispiness |
| Caramel flavored fudge candy |  | Caramel flavor |

**Table S.6.** Means of sensory attributes of replicated genotypes among samples evaluated by the trained panel in office setting

| Attribute | Genotypes | | | | P^1^ |
| --- | --- | --- | --- | --- | --- |
|  | NASPOT 11 | | MDP 452 | |  |
|  | Rep 1 | Rep 2 | Rep 1 | Rep 2 |  |
| Aroma |  |  |  |  |  |
| Sweetpotato | 7 ± 2 | 6 ± 3 | 7 ± 2 | 6 ± 2 | 0.294 |
| Pumpkin | 0 ± 0 | 0 ± 0 | 0 ± 0 | 0.3 ± 0.7 | 0.407 |
| Appearance |  |  |  |  |  |
| Orange color intensity | 0.4 ± 0.5 ^a^ | 0.4 ± 0.5 ^a^ | 3 ± 1 ^b^ | 3 ± 1 ^b^ | <0.001 |
| Uniformity of color | 7 ± 2 | 8 ± 1 | 7 ± 2 | 8 ± 1 | 0.525 |
| Degree of translucency | 2 ± 2 | 3 ± 2 | 1 ± 2 | 1 ± 2 | 0.126 |
| Fibrous appearance | 1 ± 2 | 2 ± 2 | 0.4 ± 1 | 0.4 ± 1 | 0.103 |
| Flavors |  |  |  |  |  |
| Sweetpotato | 7 ± 2 | 6 ± 2 | 7 ± 2 | 7 ± 2 | 0.904 |
| Pumpkin | 0 ± 0 | 0.3 ± 1 | 0.6 ± 1 | 0.4 ± 0.7 | 0.552 |
| Cooked carrot | 0 ± 0 | 0 ± 0 | 0.1 ± 0.4 | 0.4 ± 1 | 0.504 |
| Floral | 0 ± 0 | 0.3 ± 0.7 | 0 ± 0 | 0 ± 0 | 0.358 |
| Sweet taste | 6 ± 1 | 7 ± 1 | 6 ± 1 | 5 ± 2 | 0.407 |
| Sour taste | 0 ± 0 | 0 ± 0 | 0 ± 0 | 0.3 ± 0.7 | 0.171 |
| Bitter taste | 0.1 ± 0.4 | 0 ± 0 | 0 ± 0 | 0 ± 0 | 0.235 |
| Texture in mouth |  |  |  |  |  |
| Fracturability | 3 ± 3 ^a^ | 4 ± 1 ^a^ | 5 ± 2 ^ab^ | 7 ± 3 ^b^ | 0.028 |
| Hardness/firmness | 2 ± 2 ^a^ | 3 ± 1 ^b^ | 4 ± 1 ^bc^ | 5 ± 1 ^c^ | 0.001 |
| Crunchiness | 1 ± 1 | 1 ± 1 | 1 ± 1 | 2 ± 2 | 0.327 |
| Moisture in mass | 6 ± 2 ^b^ | 5 ± 3 ^ab^ | 4 ± 2 ^a^ | 2 ± 2 ^a^ | 0.031 |
| Crumbliness | 3 ± 2 ^a^ | 4 ± 3 ^ab^ | 6 ± 2 ^b^ | 6 ± 2 ^b^ | 0.027 |
| Fibrousness | 0.4 ± 1 | 0.9 ± 2 | 0.3 ± 0.7 | 0.1 ± 0.4 | 0.457 |
| Smoothness | 9 ± 1 ^c^ | 8 ± 2 ^c^ | 6 ± 1 ^b^ | 4 ± 2 ^a^ | <0.001 |
| Rate of breakdown | 8 ± 2 | 7 ± 2 | 7 ± 2 | 6 ± 2 | 0.611 |
| Texture by hand |  |  |  |  |  |
| Moisture release | 1 ± 2 | 2 ± 2 | 0.8 ± 2 | 0.6 ± 2 | 0.732 |
| Cohesiveness | 8 ± 1 ^b^ | 8 ± 1 ^b^ | 5 ± 3 ^a^ | 5 ± 3 ^a^ | 0.001 |
| Crumbliness by hand | 3 ± 2 ^a^ | 3 ± 2 ^a^ | 6 ± 2 ^b^ | 6 ± 1 ^b^ | 0.001 |
| Data analysis by using generalized linear models in SPSS using data of replicated genotypes only  Values in the same row with a different superscript are statistically different, p<0.05, multiple comparisons by Duncan’s Multiple Range test  Rep 1 = first replicate ; Rep 2 = second replicate  ^1^ P-value by F-test  ^2^ Genotypes evaluated in duplicate | | | | | |

**Table S.7.** Means of dry matter (%) and instrumental texture parameters of 12 genotypes of DDBIO advanced trial planted in 2020

| Genotype | Dry matter (%) | Instrumental texture parameters | | | | Secondary parameters | |
| --- | --- | --- | --- | --- | --- | --- | --- |
|  |  | Peak positive force 1 (gf) | Peak positive force 2 (gf) | Positive Area 1 (gf·s) | Positive area 2 (fg·s) | Cohessiveness | Gumminess (gf) |
|  | Mean ± SD ^1^ | | | | | | |
| D15 | 30 ± 0.8 ^abc^ | 2917 ± 636 ^abcd^ | 2325 ± 467 ^abc^ | 5415 ± 1347 ^abc^ | 1997 ± 405 ^abc^ | 0.38 ± 0.06 ^ab^ | 1086 ± 239 ^ab^ |
| D26 | 33 ± 2.0 ^de^ | 7181 ± 1382 ^f^ | 6164 ± 1398 ^f^ | 10551 ± 1388 ^e^ | 5677 ± 1444 ^f^ | 0.53 ± 0.08 ^d^ | 3908 ± 1330 ^f^ |
| Ejumula | 32 ± 1.4 ^cd^ | 4012 ± 618 ^d^ | 3342 ± 375 ^cde^ | 7507 ± 1666 ^d^ | 3402 ± 618 ^de^ | 0.46 ± 0.05 ^bcd^ | 1827 ± 245 ^bcde^ |
| NASPOT 11 | 38 ± 0.3 ^g^ | 3461 ± 650 ^bcd^ | 2785 ± 485 ^bcd^ | 6752 ± 1301 ^cd^ | 2348 ± 618 ^abc^ | 0.35 ± 0.03 ^a^ | 1206 ± 194 |
| NASPOT 8 | 34 ± 1.0 ^e^ | 5135 ± 646 ^e^ | 3901 ± 816 ^e^ | 9432 ± 1310 ^e^ | 3331 ± 791 ^de^ | 0.36 ± 0.08 ^a^ | 1855 ± 616 ^cde^ |
| NASPOT 10 O | 31 ± 1.4 ^cd^ | 2791 ± 733 ^abc^ | 2378 ± 634 ^abc^ | 4592 ± 1563 ^ab^ | 2241 ± 648 ^abc^ | 0.51 ± 0.10 ^d^ | 1401 ± 435 ^abcde^ |
| New Kawogo | 36 ± 1.3 ^f^ | 3998 ± 833 ^d^ | 2848 ± 739 ^bcd^ | 7423 ± 1903 ^d^ | 2367 ± 606 ^abc^ | 0.33 ± 0.10 ^a^ | 1308 ± 377 ^abcd^ |
| NKB105 | 29 ± 0.3 ^ab^ | 2487 ± 349 ^ab^ | 2058 ± 300 ^ab^ | 4636 ± 709 ^ab^ | 1878 ± 268 ^ab^ | 0.41 ± 0.06 ^abc^ | 1020 ± 221 ^a^ |
| NKB3 | 28 ± 0.1 ^a^ | 1942 ± 301 ^a^ | 1618 ± 296 ^a^ | 3007 ± 808 ^a^ | 1438 ± 280 ^a^ | 0.50 ± 0.10 ^cd^ | 969 ± 259 ^a^ |
| S36 | 29 ± 0.1 ^ab^ | 5705 ± 2238 ^e^ | 4116 ± 1629 ^e^ | 10379 ± 410 ^e^ | 3611 ± 1686 ^e^ | 0.34 ± 0.10  ^a^ | 2049 ± 1143 ^de^ |
| S47 | 30 ± 1.0 ^bc^ | 3839 ± 1207 ^cd^ | 3409 ± 1154 ^de^ | 5455 ± 1187 ^bc^ | 2931 ± 951 ^cde^ | 0.53 ± 0.08 ^d^ | 2096 ± 909 ^e^ |
| S97 | 29 ± 0.5 ^ab^ | 3440 ± 732 ^bcd^ | 2908 ± 544 ^bcd^ | 5139 ± 1244 ^bc^ | 2571 ± 574 ^bcd^ | 0.50 ± 0.04 ^d^ | 1730 ± 381 ^abcde^ |
| Cohesiveness calculated as the ratio of Positive Area 2 : Positive Area 1  Gumminess calculated as a product of Peak positive force 1 and cohesiveness  ^1^ Data analyzed in multivariate generalized linear models; mean separation by Duncan’s Multiple Range test; values in the same column with different letter superscripts are significantly different, p < 0.001 | | | | | | | |

**Supplementary Figures**


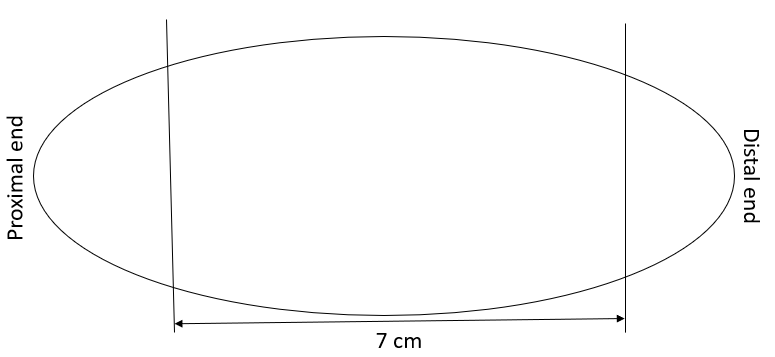


**Figure S.1.** Diagram showing how the 7 cm portions were cut from the sweetpotato root


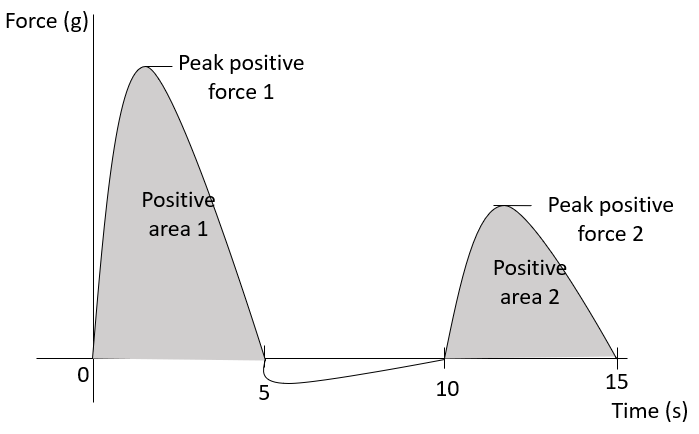


**Figure S.2.** Diagram showing parameters obtained from instrumental texture curves

**Figure S.3*.*** Principal component analysis (PCA) showing the correlation between sensory textural attributes of 6 sweetpotato genotypes (2 evaluated in duplicate) as evaluated by a trained descriptive sensory panel in the office setting

**Figure S.4.** Plot of proportion of respondents who perceived sweetpotato samples to be ‘too soft’ responses, ‘just-about-right firm’ and ‘too hard’ in on-farm trials versus average overall liking rating

**Supplementary Equations**

| $\boldsymbol{Firmness in mouth=-}24.064502296345+1.6519365607469*ln (Dry matter) +2.6114789634289* ln (Peak Force 1)$ | **(S.1)** |
| --- | --- |

**Appendices**

Appendix S.1. Consumer Questionnaire used during pilot study

CIP and its research partners are conducting research to help ensure that sweetpotato varieties introduced on the market suit consumer tastes and preferences. I will ask you a few personal questions, questions about how you eat sweetpotato. Then I will give you some sweetpotato samples to evaluate.

Participation is voluntary and you can opt out at any time.

Click the next button to begin if respondent gives consent

SECTION A: PERSONAL QUESTIONS

1. Name of respondent:

2. Gender (choose one)


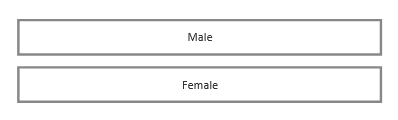


3. Date of birth:

4. Occupation (choose one)


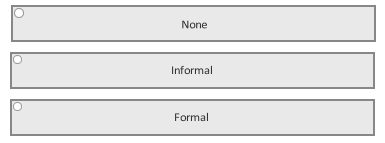


5. Marital Status (choose one)


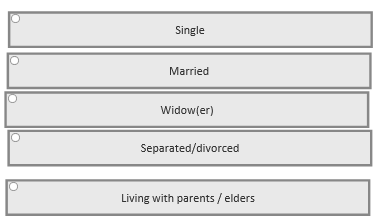


SECTION B: SWEETPOTATO CONSUMPTION PATTERNS

6. How often do you consume steamed/boiled sweetpotato? (choose 1)


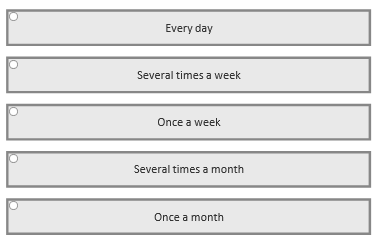


7. At what meal occasion do you consume sweetpotato the most (choose 1)


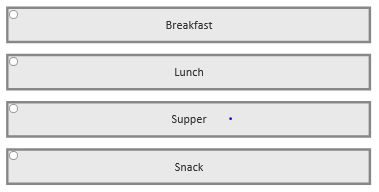


SECTION C: JUST-ABOUT-RIGHT RATING

Sample n : ###

8. Color intensity JAR

How does the intensity of the color of sample ### suit your preference? Compared to the color intensity of sweetpotato you like, is it…?


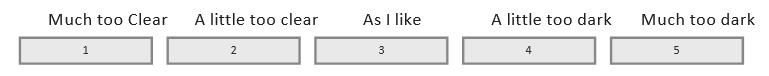


9. Sweetness JAR

How do you appreciate the SWEETNESS of sample ###? Compared to the level of sweetness you like in sweetpotato, is it…?


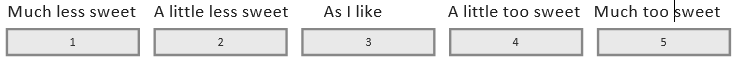


10. Firmness JAR

How do you appreciate the FIRMNESSS sample ###? Compared to the sweetpotato firmness you like, is it…?


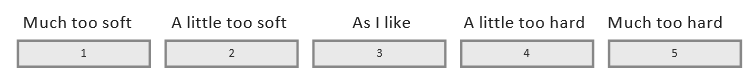


11. Mealiness JAR

How do you appreciate the MEALINESS of sample ###? Compared to the level of mealiness you like, is it …?


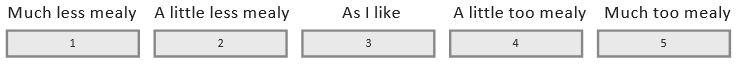


SECTION C : OVERALL LIKING RATING

12. How much do you like sample ###, overall? Do you like it…?


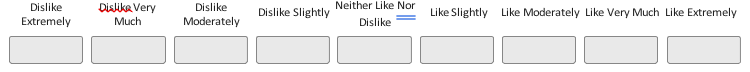


**Appendix S.2. Questionnaire – Community Consumer Testing in On-farm Trial**

Respondent ID (or questionnaire ID): …………………………………………………………………..

Name of interviewer : …………………………………………………………………….

Location of study : …………………………………………………………..

Interviewer confirmation that informed consent was given: Yes………. No……………….

**Name of participant:** ……………………………………. **Phone number:** ……………………………….

**Age category :** 18-24…….. 25-34…… 35-44……… 45-54……… 55-64 ……….. 65 and over……….

**Sex:** ………………………………………………………..

**District:** ………………………………………………………..

**Sub-County:** ……………………………………………….

**Occupation :**

- None
- Informal
- Formal

**Instructions**

**[Present the samples one at a time, ensuring that all other samples are well covered such that each sample is evaluated independently. Allow the respondent time to observe the sample by looking at it, smelling it, touching it, tasting it before you start asking the questions]**

**Sample XXX**

Sample code : …………………. [ Enter three-digit code assigned to variety ]

1a. Describe something (s) that you really like about this sample, if any. [completely open ended]

………………………………………………….

1b. Describe something(s) that you really do not like about this sample, if any. [completely open ended]

………………………………………………….

**2. Overall liking (sample ………………..)**

2a. Overall, do you like this sample or dislike this sample?

- I like it (code according to 2b.)
- I dislike it (code according to 2c)
- I neither like it nor dislike it (code = 5)

2b. If you like the sample according to 2a, how much do you like it?

- Extremely (code = 9)
- Very much (code = 8)
- Moderately (code = 7)
- Slightly (code = 6)

2c. If you dislike the sample according to 2a, how much do you dislike it?

- Extremely (code = 1)
- Very much (code = 2)
- Moderately (code = 3)
- Slightly (code = 4)

**3. Color liking (sample ………………..)**

3a. Do you like or dislike the color of this sample?

- I like it (code according to 3b.)
- I dislike it (code according to 3c)
- I neither like it nor dislike it (code = 5)

3b. If you like the sample color according to 3a, how much do you like it?

- Extremely (code = 9)
- Very much (code = 8)
- Moderately (code = 7)
- Slightly (code = 6)

3c. If you dislike the sample color according to 3a, how much do you dislike it?

- Extremely (code = 1)
- Very much (code = 2)
- Moderately (code = 3)
- Slightly (code = 4)

**4. Aroma liking (sample ………………..)**

4a. Do you like or dislike the aroma of this sample?

- I like it (code according to 4b.)
- I dislike it (code according to 4c)
- I neither like it nor dislike it (code = 5)

4b. If you like the sample aroma according to 4a, how much do you like it?

- Extremely (code = 9)
- Very much (code = 8)
- Moderately (code = 7)
- Slightly (code = 6)

4c. If you dislike the sample aroma according to 4a, how much do you dislike it?

- Extremely (code = 1)
- Very much (code = 2)
- Moderately (code = 3)
- Slightly (code = 4)

**Just About Right test (**Sample ……….**)**

5. Compared to the intensity of sweetness that you like, is the sweetness of this sample …

| Much too low | A little too low | Just as I like it | A little too high | Much too high |
| --- | --- | --- | --- | --- |
|  |  |  |  |  |

6. Compared to the intensity of firmness of sweetpotato that you like, is this sample …

| Much too soft | A little too soft | Just as I like it | A little too firm | Much too firm |
| --- | --- | --- | --- | --- |
|  |  |  |  |  |

7. Compared to the powdery texture of sweetpotato that you like, is this sample …

| Much too waxy | A little too waxy | Just as I like it | A little too powdery | Much too powdery |
| --- | --- | --- | --- | --- |
|  |  |  |  |  |
